# Environmental Enteropathy in Undernourished Pakistani Children: Clinical and Histomorphometric Analyses

**DOI:** 10.4269/ajtmh.17-0306

**Published:** 2018-04-02

**Authors:** Sana Syed, Sunil Yeruva, Jeremy Herrmann, Anne Sailer, Kamran Sadiq, Najeeha Iqbal, Furqan Kabir, Kumail Ahmed, Shahida Qureshi, Sean R. Moore, Jerrold Turner, S. Asad Ali

**Affiliations:** 1 Department of Paediatrics and Child Health, Aga Khan University, Karachi, Pakistan;; 2 Department of Pediatrics, University of Virginia, Charlottesville, Virginia;; 3 Department of Pathology, Brigham and Women’s Hospital, Harvard Medical School, Boston, Massachusetts;; 4 Department of Medicine, Brigham and Women’s Hospital, Harvard Medical School, Boston, Massachusetts;; 5 Boston Children’s Hospital, Harvard Medical School, Boston, Massachusetts;; 6 Department of Pathology, University of Chicago, Chicago, Illinois

## Abstract

Despite nutrition interventions, stunting thought to be secondary to underlying environmental enteropathy (EE) remains pervasive among infants residing in resource-poor countries and remains poorly characterized. From a birth cohort of 380 children, 65 malnourished infants received 12 weeks of nutritional supplementation with ready-to-use therapeutic food (RUTF). Eleven children with insufficient response to RUTF underwent upper endoscopy with duodenal biopsies, which were compared with U.S., age-matched specimens for healthy, celiac disease, non-celiac villous atrophy, non-celiac intraepithelial lymphocytosis, and graft-versus-host disease patients. Of the 11 children biopsied, EE was found in 10 (91%) with one subject with celiac disease. Morphometry demonstrated decreased villus-to-crypt (V:C) ratios in EE relative to healthy and non-celiac lymphocytosis patients. Environmental enteropathy villus volumes were significantly decreased relative to healthy controls. In EE, average CD3^+^ cells per 100 epithelial cells and per 1,000 µm^2^ of lamina propria and the number of lamina propria CD20^+^ B-cell aggregates were increased relative to all other groups. Our results indicate that V:C ratios are reduced in EE but are less severe than in celiac disease. Environmental enteropathy intraepithelial and lamina propria T lymphocytosis is of greater magnitude than that in celiac disease. The increases in lamina propria B and T lymphocytes suggest that non-cytolytic lymphocytic activation may be a more prominent feature of EE relative to celiac disease. These results provide new insights into shared yet distinct histological and immunological features of EE and celiac disease in children.

## INTRODUCTION

Environmental enteropathy (EE) is a malabsorptive condition that is highly prevalent in residents of low- and middle-income countries. Although EE has been linked to poor sanitation and hygiene, the underlying pathophysiology has not been elucidated.^1–5^ This may explain the wide variety of definitions and nomenclature, including tropical enteropathy, environmental enteric dysfunction, and subclinical malabsorption,^5,6^ which have been applied to EE. Nevertheless, EE is increasingly recognized as a key factor underlying malnutrition, deficient immune responses, and impaired cognitive development in children within low- and middle-income countries.^7^

The earliest studies describing enteropathy with the use of intestinal biopsies were from the 1960s to 1970s^8–10^ with study subjects from Asia, Africa, the Indian subcontinent, and Central America. These studies also reported morphological changes or functional signs of EE in a high proportion of apparently healthy adults and children.^11–14^ Functional measures included clinical or subclinical malabsorption of macro- and micronutrients, as well as increased permeability to small molecules. Despite significant published reports from the 1970s to 1980s, there was minimal focus on the underlying pathophysiology of EE. Environmental enteropathy has become an active area of investigation over the last decade. Results of both investigative analyses and clinical trials have, however, been disappointing, with nutritional interventions failing to improve growth, reduce susceptibility to infection, and increase oral vaccine responses.^15^

Given the financial and operational limitations of safely obtaining intestinal biopsies from very young children in the community, there have been very few detailed investigations of human intestinal tissue in this vulnerable patient group in whom reversal of EE would provide the greatest benefit. Previous studies have characterized the histopathology of EE as ranging from normal villi to villous shortening, crypt hyperplasia, loss of brush-border enzyme expression, and lamina propria lymphocytic and plasma cell infiltrates.^4,5,16–18^ Recently, several groups have endeavored to establish animal models of EE using low-protein diets in combination with perturbation of the intestinal microbiota.^19,20^ Although villous blunting and altered epithelial and mucosal immune responses were induced, the extent to which these models recapitulate human disease is difficult to assess, given the paucity of studies characterizing tissue from human EE cases. Hence, the aims of the current study, thus, were to define the histopathologic changes in the small intestine in well-characterized EE cases and to compare these findings with U.S. healthy and selected disease controls.

## MATERIALS AND METHODS

### Study design and participants.

Subjects included in this analysis were part of a prospective community-based active surveillance birth cohort with an intervention arm that was designed to investigate growth faltering, response to ready-to-use therapeutic food (RUTF), and endoscopic evaluation of children with an inadequate growth response to RUTF. The study site was in the rural district of Matiari, located in the province of Sindh about 200 km (124 miles) north of Karachi, Pakistan. The Department of Pediatrics and Child Health at the Aga Khan University, Pakistan, has established research infrastructure and relationships in this area in collaboration with the Government of Pakistan since 2002 for the purpose of community-based research.

Enrollment and assessment of newborns were carried out during routine surveillance of pregnant women of reproductive age (13–49 years) by community health workers (CHWs). Study inclusion criteria were 1) newborns aged up to 14 days; 2) absence of any major congenital abnormalities; and 3) ability to obtain informed consent from parents or guardians. Infants of families planning to move out of the study area within 6 months of birth were excluded from the trial. Enrolled children were followed from birth (0–14 days) until 18 months of age with weekly home visits. At each weekly visit, CHWs obtained morbidity data, including number of days of reported fever, symptoms of acute respiratory infections (ARI), vomiting, and diarrhea in the preceding week. Days and types of antibiotics received were recorded at monthly visits for each of the 10 EE biopsy subjects. However, none of these subjects received antibiotics in the 4–6 weeks before biopsy. All families enrolled in the study were provided with cell phone contact information of key study physicians to enable direct and immediate contact in the case of any urgent medical need by the study participants.

Monthly measurements were recorded by trained CHWs using standard techniques: child’s weight using a digital infant balance with 20-g precision (TANITA 1584) and the child’s length with 1-mm precision (using a rigid length board with a movable foot piece). Standardization of measurements was ensured through regular staff training and cross-checks.

### Sample section of subjects and controls.

Children who were identified as having moderate (weight-for-height *z* score [WHZ] < −2) or severe (WHZ < −3) acute malnutrition at 9 months of age were selected for intervention with educational counseling and energy dense nutrition supplementation (Supplemental Figure 1). Educational intervention was provided over a 2-month period of weekly home visits. Teaching material covered included benefits of breastfeeding, maternal diet, and age-appropriate complementary feeding and was delivered via interactive sessions between the parents and caregivers using standardized training videos. Nutritional intervention included provision of weight appropriate quantity of RUTF^21^ (F-100 formula, 500 kcal/92 g sachet) for 2 months, with close monitoring of compliance (defined as the proportion of sachets consumed of the total prescribed sachets that were supplied) which was monitored weekly. Upper gastrointestinal endoscopies were performed for a subset of children who did not respond to RUTF. Control subjects were selected from the tissue archives of the Department of Pathology at the University of Chicago. Duodenal biopsies from 52 age-matched controls were selected and divided into five distinct groups: healthy normal (25), celiac disease (7), non-celiac villous atrophy (7), non-celiac intraepithelial lymphocytosis (8), and graft-versus-host disease (2).

Of note, all intestinal tissue samples from the Aga Khan University, Pakistan, were from our study selection process as outlined previously and not from archives. Paraffin-embedded duodenal biopsies were obtained from the Aga Khan University, Pakistan, from our 10 study subjects. Archival tissue from the University of Chicago Department of Pathology was used as outlined previously as controls.

### Assessment of micronutrient and celiac disease status.

Blood was collected from all subjects at the time of endoscopy in a venous blood collection tube coated with EDTA (Becton Dickinson, Franklin Lakes, NJ) and in a 3–5-mL serum gel yellow-top vacutainer (Becton Dickinson) at 10 months of age to screen for micronutrient deficiencies and celiac disease. Whole blood samples of 3–5 mL each were immediately sent for the following laboratory tests per standard clinical protocol: complete blood count for hemoglobin (Hb), white blood cell (WBC) count, and cell differential (SYSMEX XE 5000; Sysmex Corporation, Kobe, Japan). Plasma was obtained by centrifuging the BD gel vacutainer (Becton Dickinson) according to the manufacturer’s specifications and then aliquoted and stored at −80°C. The following assays were run on the serum samples: ferritin (Immulite 2000; Siemens, Erlangen, Germany); B12 concentrations (ADVIA Centaur; Siemens); transferrin saturation (TSat) % (ADVIA Centaur XP; Roche, Erlangen, Germany); folic acid (ADVIA Centaur; Siemens); and 25-hydroxy (OH) vitamin D (LIASON XL; DiaSorin, Saluggia, Italy). Celiac screening investigations consisted of anti-tissue transglutaminase immunoglobulin A (tTG IgA) (ETIMAX 3000; DiaSorin, ETIMAX, enzyme-linked immunosorbent assay) and total serum IgA (ADVIA 1800; Siemens).

Biomarkers were defined as abnormal according to the following cutoffs: anemia, Hb < 11.0 g/dL guidelines^22^; leukocytosis, WBC Count > 18.0 × 10^3^ μL (per clinical laboratory manual); iron deficiency, serum ferritin < 15 µg/L or TSat% < 20^23^; B12 deficiency, serum B12 < 150 pg/mL^24^; folate deficiency, serum folic acid < 3 ng/mL^25^; vitamin D deficiency, 25(OH)D < 20 ng/mL,^26^ vitamin D insufficiency, 25(OH)D levels between 20 and 30 ng/mL^26^; anti-tTG-IgA: < 12 U/mL = negative, 12–18 U/mL = equivocal, and > 18 U/mL = positive (per clinical laboratory manual); and total serum IgA = normal range 0.18–1.7 g/L (per clinical laboratory manual).

### Endoscopy and tissue handling.

The proximal intestinal mucosa was biopsied endoscopically by a trained pediatric gastroenterologist using standard Olympus Evis Exera III CV-190 pediatric endoscope, which has a working channel that allows a 2.8-mm biopsy forceps. Gastric biopsies were obtained only if the mucosa looked endoscopically abnormal in *N* = 8 of the total 10 subjects. Duodenal biopsies were obtained for all children using standard biopsy forceps and immediately fixed in 10% neutral buffered formalin and processed for embedding in paraffin.

### TaqMan array card (TAC).

The TaqMan low-density array card or TAC allows molecular detection of multiple pathogens using a customized detection platform for common enteric pathogens^27^ that included viruses, bacteria, and helminths. The TAC card was used to detect microbial targets in duodenal aspirates and stool of the *N* = 10 biopsy subjects. This platform has been validated previously in different laboratories in international multicenter studies.^28^ Briefly, total nucleic acid (TNA) was extracted from 400 μL of duodenal aspirates run through an automated magnetic bead–based extractor, using Roche MagNa Pure Compact isolation kit I (Roche Life Sciences, Mannheim, Germany). All duodenal aspirates were spiked with internal controls of phocine herpes virus (PhHV) and MS2 (MS2 bacteriophage) as DNA and RNA targets, respectively, for validation and efficiency of extraction, reverse transcription, and amplification steps. The TAC protocol was modified from that of the Next Gen project (Houpt Laboratory, University of Virginia, Charlottesville, VA). A total of 100 μL of TNA was eluted from the MagNa Pure analyzer and 40 μL of TNA was loaded on a TAC card through microfluidic ports. The card was sealed and run on ViiA7 (Applied Biosystems, Thermo Fisher, Waltham, MA). A total of eight samples were run on a single card along with extraction blanks (consisting of nuclease-free water with PhHV and MS2), polymerase chain reaction (PCR) blank (consisting of nuclease-free water only), and TAC-mixed positive controls. The samples were considered a valid positive if 1) the sample’s target cycle threshold value was less than 32, 2) the reference extraction blank was negative for each target, and 3) the internal control had a cycle threshold value less than 3.

### Intestinal tissue handling and hematoxylin and eosin (H&E).

Paraffin-embedded duodenal biopsies were obtained from the Aga Khan University in Pakistan and the University of Chicago Department of Pathology archives. *Helicobacter pylori* was identified using routine H&E staining. De-identified biopsies archived at the University of Chicago of children with histopathologic diagnoses of 1) non-celiac villous atrophy, 2) non-celiac intraepithelial lymphocytosis, and 3) normal were used as comparative groups in our study. These were children who had underwent endoscopies for a variety of clinical indications, including (but not limited to) evaluation of failure to thrive and/or diarrhea with serology, which was negative for celiac disease. These subjects were classified as follows based on the appearance of their duodenal mucosa: healthy normal, celiac, graft-versus-host-disease (GVHD), non-celiac villous atrophy, and non-celiac lymphocytosis. Please see Supplemental Tables 1–3 for details of these clinical indications. After examination of clinical records, pathology reports, and H&E–stained slides, the University of Chicago cases were subdivided into normal, non-celiac lymphocytosis, non-celiac villous atrophy, celiac disease, and graft-versus-host disease cases.

### Morphometry for villus and crypt measurements and immunohistochemical staining.

Measurement of villus height and crypt depth was performed on the H&E–stained slides. The slides were scanned using Leica DM4000 microscope using a 20×/NA0.7 PLAN-APO objective, Photometrics^®^ CoolSNAP HQ2 camera for fluorescent images and ProgRes^®^ C10plus from Jenoptik for bright-field images, and the slidescan feature using custom journals within Metamorph 7 (Molecular Devices, San Jose, CA). Only well-oriented crypt–villus units within each section were measured. Distances from the villus tip to crypt–villus junction and to the crypt base were measured using Metamorph. Villus volume was calculated by modeling the villus as a cone. Villus width, that is, diameter, was measured at 1/3 and 2/3 of the villus height. Villus-to-crypt (V:C) ratios and villus volume could not be determined for graft-versus-host disease cases because of the absence of representative villi in these biopsies.

Intraepithelial lymphocyte numbers were labeled by anti-CD3 staining. The slides were baked for 90 minutes at 60°C, washed in xylene three times for 15 minutes, transferred to 100% ethanol, and rehydrated to water through a series of ethanol solutions. Antigen retrieval was performed for 40 minutes using Dako Target Retrieval Solution (S2367) in a steamer. After three washes in tris-buffered saline tween (TBST) (50 mM Tris, pH 7.5, with 0.1% tween-20), incubation for 20 minutes in 50 mM NH_4_Cl in tris-buffered saline (TBS) (50 mM Tris, pH 7.5), three subsequent washes with TBS, and blocking solution with 0.3 M glycine, 5% bovine serum albumin, 10% normal donkey serum, and 10% normal human serum in TBS, the slides were incubated with antibodies against CD3 (#ab16669; Abcam, Cambridge, United Kingdom) and CD20 (#ab9475; Abcam) diluted in blocking solution for 24 hours at 4°C. The slides were then washed three times in TBST and twice with TBS before applying AlexaFluor 488 AffiniPure F(ab′)_2_ donkey anti-mouse immunoglobulin G (heavy and light chains) (#715-546-150; Jackson ImmunoResearch, West Grove, PA), AlexaFluor 594 AffiniPure F(ab′)_2_ fragment donkey anti-rabbit (#711-586-152; Jackson ImmunoResearch), and Hoechst 33342 (1 μg/mL) for 2 hours at room temperature. After six washes in TBST, the slides were dipped in water and mounted using ProLong Gold antifade (Thermo Scientific). Imaging was performed using a Leica DM4000 microscope configured as aforementioned using a Photometrics CoolSNAP HQ2 camera.

### Data management and statistical analysis of intestinal tissue.

The World Health Organization (WHO) Child Growth Standards (WHO Anthro, Geneva, Switzerland) were used to calculate *z* scores and categorized stunting as a height-for-age *z* score < −2 standard deviation (SD), underweight as a weight-for-age *z* score < −2 SD, and wasting as a WHZ < −2 SD. Scanned images of anti-CD3 and anti-CD20 immunostains were scored using the threshold function of Metamorph to assess fluorescence intensity. Cells that exceeded threshold limits were counted as applied, and biopsies were deemed to be positive. Analysis of variance and *t* tests were performed for all morphometric statistical analyses using GraphPad Prism version 6.00 for Windows and GraphPad Software (La Jolla, CA, www.graphpad.com). SAS 9.3 (SAS Institute Inc., Cary, NC) was used to calculate the chi-squared test and compare B-cell aggregate numbers between different groups.

### Ethics statement and study data.

Institutional approval was granted by the Aga Khan University Human Subjects Committee and the University of Chicago Institutional Review Board. Data from this study are not available in a public repository but will be made available on request.

## RESULTS

### Background characteristics of EE biopsy subjects.

Of the 380 children who were enrolled at birth between November 2012 and April 2013, 65 children had WHZ < −2 and met the eligibility criteria to receive 12 weeks of intensive nutritional (RUTF) intervention and educational counseling. Table 1 and Supplemental Table 4 summarize the background characteristics of our study cohort of *N* = 10 EE cases. Maternal age on average was nearly 27 years with no mothers having had any formal education; half had their delivery at home. Fifty percent (*N* = 5) of all subjects had low birth weight (birth weight < 2,500 g) and *N* = 6 children (60%) were born preterm (gestational age < 37 weeks), and all the children were breastfed. As noted in Supplemental Figure 2, recurrent episodes of ARI and diarrhea were a predominant feature with the average number of diarrheal days per month ranging from 0 to 8 days, whereas the average number of ARI days per month ranged from 0 to 25 days. The highest average WHZ score was −0.70, reaching −2.6 just before biopsy at 18 months of age.

**Table 1:** Characteristics at the time of biopsy from EE cases and Western controls

	Total population median (Q1–Q3) or *n* (%)
Diagnosis	
Normal	25 (42%)
EE	10 (17%)
Celiac disease	7 (12%)
Lymphocytosis	8 (14%)
Villous atrophy	7 (12%)
Graft vs. host disease	2 (3%)
Age, months*	
Normal	12 (12–12)
EE	22 (20–23)
Celiac disease	12 (12–12)
Lymphocytosis	12 (12–12)
Villous atrophy	10 (2–12)
Graft vs. host disease	30 (12–48)
Gender	
Female	29 (49%)
Race	
African American	9 (15%)
Asian	2 (3%)
South Asian	10 (17%)
Hispanic	2 (3%)
Caucasian	27 (46%)
Unknown	12 (20%)

Total patients *N* = 59; 61 biopsies obtained from *N* = 49 Western control patients, 27 biopsies obtained from *N* = 10 EE biopsy cases. EE = environmental enteropathy.

*Nonparametric comparisons were made between the age of EE subjects and normal, celiac disease, lymphocytosis, villous atrophy, and graft vs. host disease subjects; age was significantly different (*P* < 0.05) in all group comparisons except EE vs. GVHD.

### Micronutrient status, investigations, and PCR microbial detection summary of EE biopsy subjects.

Anemia was present in *N* = 7 (70%) of the children with 20% (*N* = 2) with leukocytosis (Supplemental Table 4). Almost all children *N* = 9 (90%) had iron deficiency, one had B12 deficiency, and of the folate status measured, all (*N* = 9) were normal. Of the *N* = 9 children who had plasma 25(OH)D levels measured, only one child was vitamin D sufficient, with the rest being either deficient (*N* = 3) or insufficient (*N* = 5). Celiac screening was performed using serum tTG-IgA, which was negative in nine subjects and equivocal in one (note: of our total 11 children biopsied, EE was found in 10 [91%] with one subject with celiac disease [anti-transglutaminase-IgA level of 97.32 U/mL]). All had normal IgA levels. Of the 10 duodenal aspirate samples collected, one child was both *Giardia* spp. and *H. pylori* positive, one child had *Enterovirus*, *Giardia* spp., and *H. pylori* positive, and finally, one child had *Campylobacter* (*jejuni* and *coli*), *Giardia* spp., and *Norovirus* GII positive. *Helicobacter pylori* and *Giardia* spp. were the most common pathogens, being positive in four patients each (Supplemental Table 5). No stool testing was carried out at clinical presentation or at the time of endoscopy; however, pathogens were tested in stools at the age of 6 and 9 months using the same TaqMan array as aforementioned in *N* = 10 study subjects (parental refusal for one subject). These have been summarized in Supplemental Table 6.

### Clinical characteristics.

Biopsies were available for further analysis from a total of 59 children with the following diagnoses: normal Western healthy controls (25), EE (10), Western celiac disease (7), Western non-celiac lymphocytosis (8), Western non-celiac villous atrophy (7), and Western graft-versus-host disease (2). Median (Q1 and Q3) ages of subjects at the time of biopsy ranged from 10 (2–12) months (villous atrophy subjects) to 30 (12–48) months (GVHD subjects); EE cases were 22 (20–23) months. The subjects were approximately equally divided between males (51%, *N* = 30) and females (49%, *N* = 29).

### Degrees of villous blunting and intraepithelial lymphocytosis differ between EE and other diseases.

**Figure 1.**
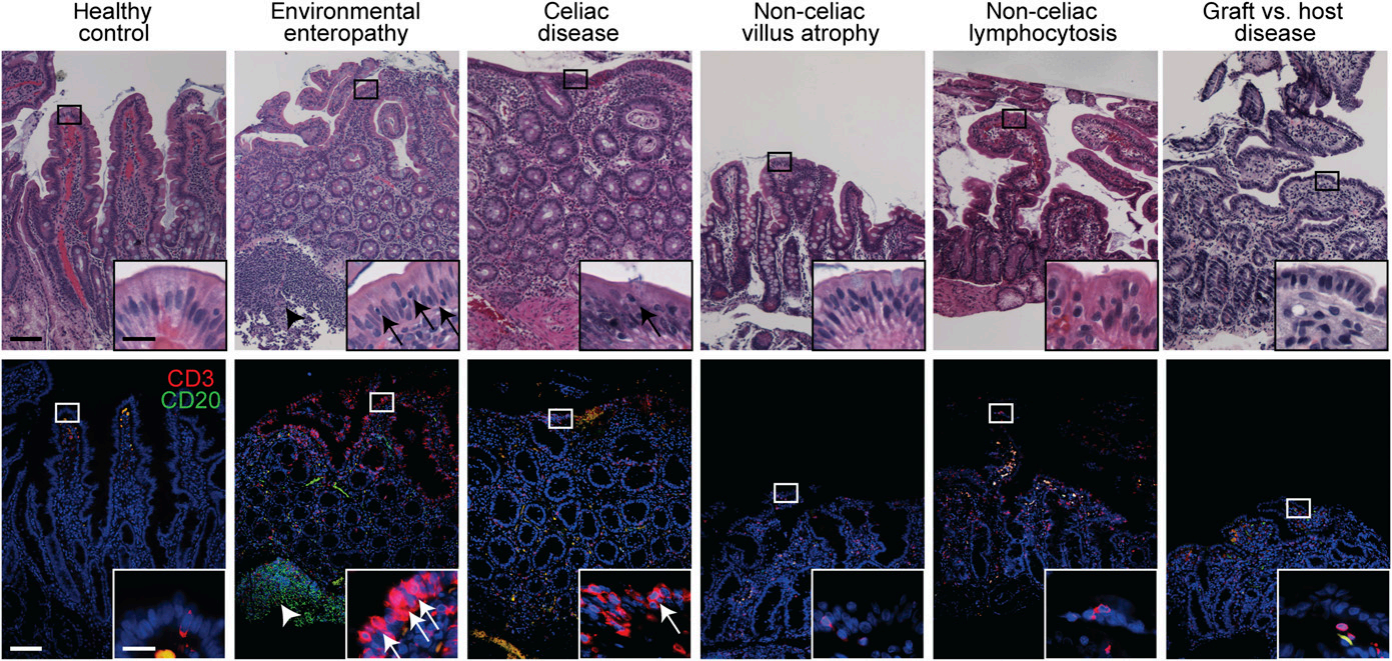
Duodenal histology and immunohistochemistry in environmental enteropathy (EE). Representative hematoxylin and eosin (H&E) (top) and immunofluorescence (bottom) images of matched fields for each subject group are shown. Immunofluorescence shows T-cell marker CD3 (red), B-cell marker CD20 (green), and a DNA stain (blue). Boxed areas on low magnification images designate the area shown in the inset. Arrows in the immunofluorescence images designate T cells. The arrowhead in the immunofluorescence image of the EE case indicates a lymphoid aggregate. Scale: low magnification images, bar = 100 μm; insets, bar = 20 μm. The H&E fields shown are of the same regions shown in the immunofluorescence images. These were selected to best demonstrate the immune infiltrates. In the cases of EE and GVHD, well-oriented crypt–villus units were not present in the most illustrative immunostained regions.

**Figure 2.**
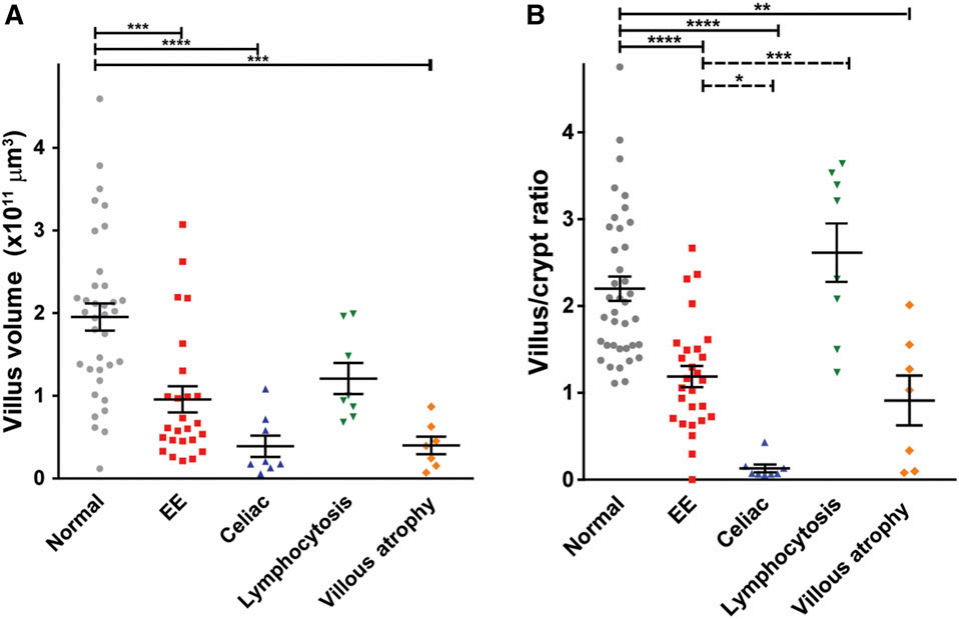
Villus and crypt morphometry in environmental enteropathy (EE). (**A**) Villus volume was calculated by using villus depth and villus width at 1/3 and 2/3 from the tip of the villus from the biopsies of healthy normal, EE, lymphocytosis, celiac disease, and villous atrophy biopsies. (**B**) Villus-to-crypt ratios were measured in healthy normal, EE, lymphocytosis, celiac disease, villous atrophy, and GVHD patient biopsies. Note: one-way analysis of variance with Bonferroni’s multiple comparison tests was performed with horizontal bars indicating significant results. ****P value < 0.00005; ***P value < 0.0005; **P value < 0.005; *P value < 0.05.

Villus height and crypt depth, which indicate damage and regenerative responses, respectively, are often presented as V:C ratio. The V:C ratio of EE cases was significantly reduced in EE cases relative to healthy Western controls (Figures 1 and 2). This was due to both modest villous blunting and crypt hyperplasia. The degree to which V:C ratio was reduced in EE was comparable to that in western cases of non-celiac villous atrophy, but far less than the total villous atrophy observed in western celiac disease cases within this age group. Consistent with loss of villus height and reduced V:C ratio, villus volumes were also significantly decreased in EE but were affected more severely in Western celiac disease (Figure 2).

**Figure 3.**
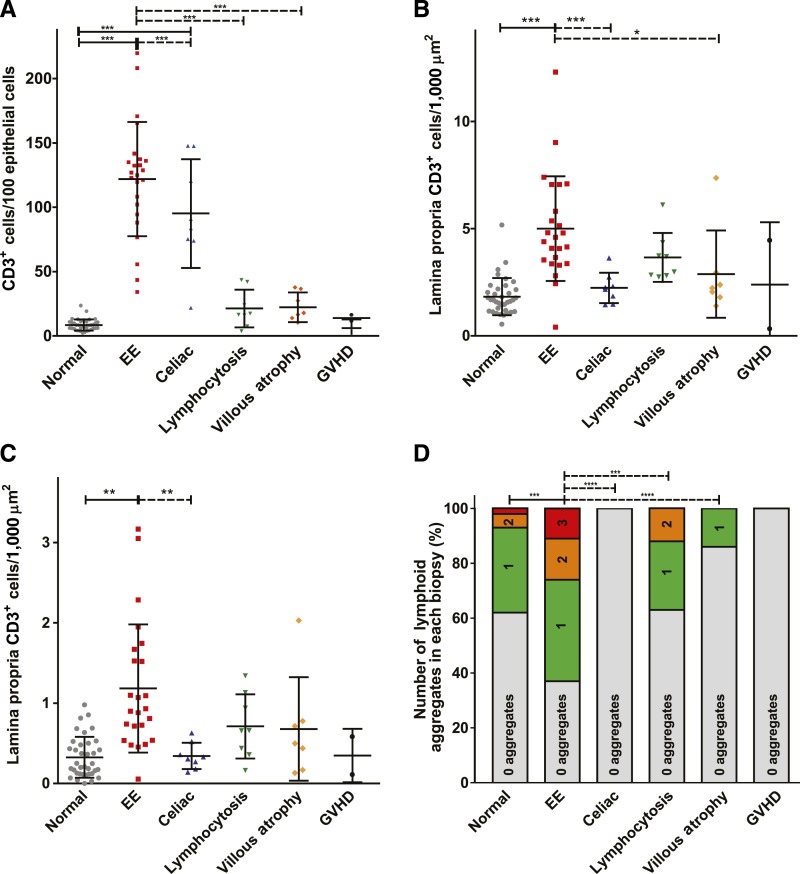
Intraepithelial and lamina propria lymphocyte populations in samples from patients with healthy normal, environmental enteropathy (EE), lymphocytosis, celiac disease, villous atrophy, and GVHD patient biopsies. (**A**) Intraepithelial CD3^+^ T lymphocytes. (**B**) Lamina propria CD3^+^ T lymphocytes. (**C**) Lamina propria CD20^+^ B lymphocytes. (**D**) Bar graphs demonstrating percentages of CD20^+^ B lymphocyte aggregates. For (**A**–**C**), one-way analysis of variance with Bonferroni’s multiple comparison tests was performed with horizontal bars indicating significant results. For (**D**), χ^2^ comparisons were performed with horizontal bars indicating significant results. ***P value < 0.0005; **P value < 0.005; *P value < 0.05.

Increased numbers of intraepithelial T lymphocytes, particularly within villous epithelium, is a well-characterized feature of celiac disease and was present in the Western celiac disease cases analyzed in this study (Figures 1 and 3A and B). Previous studies have suggested that, as with villous atrophy, the intraepithelial lymphocytosis in EE is similar to that occurring in celiac disease. However, the cases analyzed here show that increased intraepithelial lymphocytosis in EE is more intense than that in all other conditions including celiac disease (Figures 1 and 3A). Lamina propria T-cell infiltrates in EE were also far denser than those in other conditions (Figures 1 and 3B). Thus, although EE histopathology is qualitatively similar to celiac disease, when analyzed in quantitative terms, EE displays less severe epithelial damage and far more intense T-cell infiltrates within both the intraepithelial and lamina propria compartments.

### Mucosa-associated lymphoid tissue induction distinguishes EE from celiac disease.

Our analyses showed that lymphoid follicles with germinal centers (Figure 1) were common in EE biopsies relative to healthy controls and other diseases. When quantifying numbers of lymphoid aggregates per biopsy, our results were notable for more than 60% of EE biopsies including one of more lymphoid aggregates, with 25% having two or three aggregates per biopsy. We also quantified numbers of lymphoid aggregates per disease group (normal, EE, non-celiac villous atrophy, and non-celiac lymphocytosis)—of note, 40% of healthy controls and less than 40% of non-celiac lymphocytosis cases had lymphoid aggregates. Moreover, few of these had more than one follicle per biopsy fragment. These differences were significant when EE cases were compared with any of the other groups (*P* < 0.0001) (Figure 3D). Statistical analysis (two-sided Fisher’s exact test) confirmed that in the case of *H. pylori*, pathogen positivity and presence of ≥ 2 lymphoid aggregates did not co-occur more frequently than as expected by chance (*P* value not significant). In the case of *Giardia*, pathogen positivity and presence of ≥ 2 lymphoid aggregates co-occurred more frequently than as expected by chance (*P* value < 0.05) (Supplemental Table 7).

## DISCUSSION

The goal of this investigation was to define the duodenal histopathology of EE in a well-characterized population of rural Pakistani infants and to compare these findings with healthy and disease controls. Our EE cases were typical in that they were typified by maternal illiteracy, infant low birth weight/preterm status, and multiple micronutrient deficiencies. We found that V:C ratios among EE cases were significantly different from controls with celiac disease or non-celiac lymphocytosis. In addition, although both were characterized by increased numbers of intraepithelial T cells, the magnitude of these increases in EE was greater than that in celiac disease. Finally, EE cases had increased lamina propria T-cell infiltrates and B-cell aggregates, consistent with broader and more intense lymphocytic recruitment than in celiac disease or any of the other control categories. This may be related to altered microbiota, as more than half of the EE cases had microbial overgrowth as indicated by positive duodenal aspirate microbial testing.

Prior studies describing the histopathologic features of the gut mucosa have also been performed in children with a variety of clinical presentations and sites. For example, Sullivan et al.^29^ reported on jejunal biopsies from severely malnourished Gambian children, all of whom had either kwashiorkor or marasmus. Conversely, the Gambian children studied by Campbell et al.^18^ were heterogeneous, with a wide range of nutritional and clinical states (median weight *z* score, −4.6; range, 0.5 to −6.4). Finally, studies in Brazil evaluated asymptomatic children with altered D-xylose absorption and, on biopsy, moderate villous atrophy with lamina propria inflammation. Here, we have sampled a homogenous group of children, all with WHZ < −2, who had all failed RUTF nutritional intervention. As a result, the features we identified in these biopsies are more likely to be specific for EE rather than across the range of malnutrition and malabsorptive disorders seen in impoverished populations.

Previous studies generalized the histopathology of EE as being identical to celiac disease.^29,30^ However, even when compared with our age-matched celiac disease cases, the EE cases displayed more intense intraepithelial T lymphocytosis. This is remarkable, given that all of the celiac disease cases had complete villous atrophy, whereas villi were relatively preserved in EE.

A previously unrecognized observation is the number of lamina propria B lymphocyte aggregates in the EE biopsies we evaluated. Of 27 biopsies from 10 EE patients, all except one case had at least one B-cell aggregate identified. This was significantly greater than that seen in celiac disease or other patient categories evaluated and suggests that B-cell aggregates are common enough to consider a “typical” feature of EE. It remains to be determined whether the presence of B-cell aggregates, lamina propria T-cell infiltrates, and intraepithelial T lymphocytosis correlates with the magnitude of the microbial burden or specific microbial changes in EE. Alternatively, the increased immune cell recruitment could be an adaptive response to inadequate specific activation of mucosal immunity. Consistent with this, we have recently shown that serum levels of anti-flagellin and anti-lipopolysaccharide IgA were significantly lower in our cohort of Pakistani infants than in healthy infants in Boston.^31^ Similarly, Kau et al.^32^ have reported a relationship between IgA responses and growth in two cohorts of Malawian infants and children.

There are several limitations to this study. First, we did not measure detailed metabolic profiles of our subjects at the time of endoscopy. Second, the criteria we used to triage and identify which children needed to get endoscopies included the use of WHZ scores < −2 and clinical history concerning malabsorption (history of recurrent diarrhea, abdominal bloating, etc.), which were broader than those in other prior studies in community settings in which children were biopsied. As noted in our methods, days and types of antibiotics received were recorded at monthly visits for each of the 10 EE biopsy subjects; however, we believe that the modest number of patients, the large variety of pathogens, and the diversity of antimicrobials administered would make a definitive analysis difficult; thus, these data were not included in our present work. The nonindependence of biopsy measures per subject is a potential limitation and has been included as such in the limitations. As noted before, these are results from a preliminary study, which we have used to inform a currently ongoing better powered investigation with larger sample size. An important point to note would be that the age between our normal controls and EE subjects was significantly different; however, present literature would suggest that dimensions in healthy normal intestinal villi and crypts change with age such that mean crypt length is longer in infants with significantly less mean epithelial cell height.^33,34^ This would suggest in our groups, the younger “normal” group would have V:C ratios lower than an older “normal” group, suggesting if anything our results have likely underestimated the true effect size. This has been noted in our discussion also. Finally, maternal data were relatively limited, the sample size was small, and in-country controls were not available. Nevertheless, our study had important strengths including the relative homogeneity of our subjects, use of age-matched normal and disease controls, and detailed qualitative and quantitative analyses of immune cell infiltrates using immunofluorescence microscopy, which provides greater spatial resolution than enzymatic immunohistochemical methods. Furthermore, although WHZ was our primary criterion for selection of children for nutritional intervention, children were selected for biopsy if they showed a clinical concern for malabsorption and/or ponderal and linear growth faltering, as shown in our results. Because of the overlap in children who were underweight, wasted, and stunted, we feel our results are relevant to the height-for-height *z* score (HAZ) drop that is seen in EE. Indeed, the 10 EE children biopsied in our study experienced an average HAZ drop of two *z* scores from birth to 22 months of age. Finally, we have demonstrated the safety and ethical and practical feasibility of performing endoscopic evaluation of malnourished children in the community setting.

In summary, among a birth cohort of rural Pakistani children at risk of developing EE, we have demonstrated in a subset of malnourished children that multiple micronutrient deficiencies, infections, and characteristic histopathologies coexist. These findings have important implications both in our understanding of EE and in developing a clinical case definition along with identifying future areas that deserve further research. As we come closer to truly understanding the pathophysiology of gut function among malnourished children living in resource-poor countries, we narrow the gap toward effective treatments both for improving oral vaccine response and stunting in these settings.

## Supplementary Material

Supplemental Tables and Figures
