# Supplementary material for: Environmental Enteropathy in Undernourished Pakistani Children: Clinical and Histomorphometric Analyses
Source: Am J Trop Med Hyg. 2018 Apr 2;98(6):1577–84. doi: 10.4269/ajtmh.17-0306 (PMC6086170; doi:10.4269/ajtmh.17-0306)
Supplement: Supplementary file 1 [file tpmd170306.SD1.pdf]

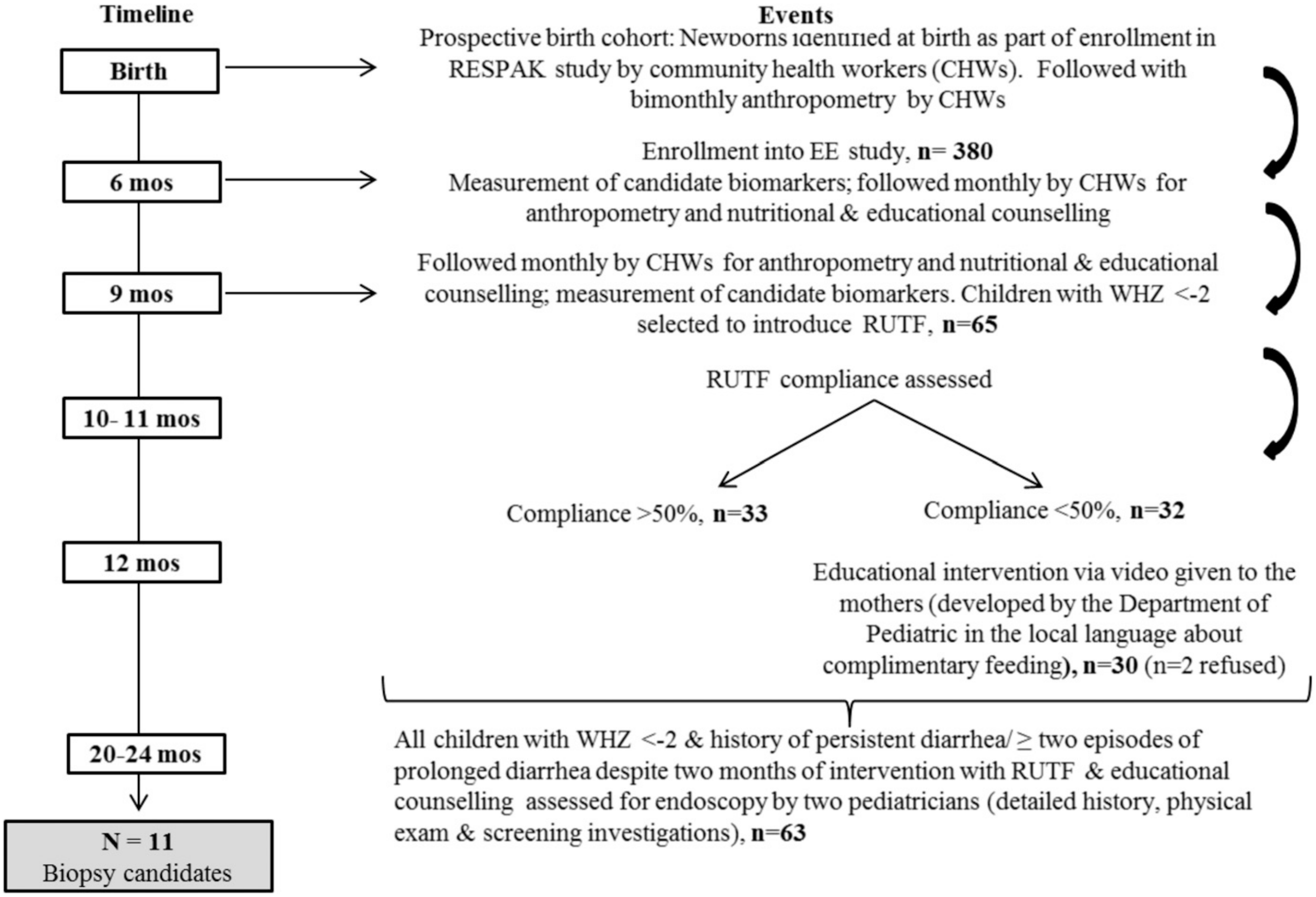

Supplemental Figure 1. Flow of study participants during study period outlining selection of environmental enteropathy biopsy candidates.

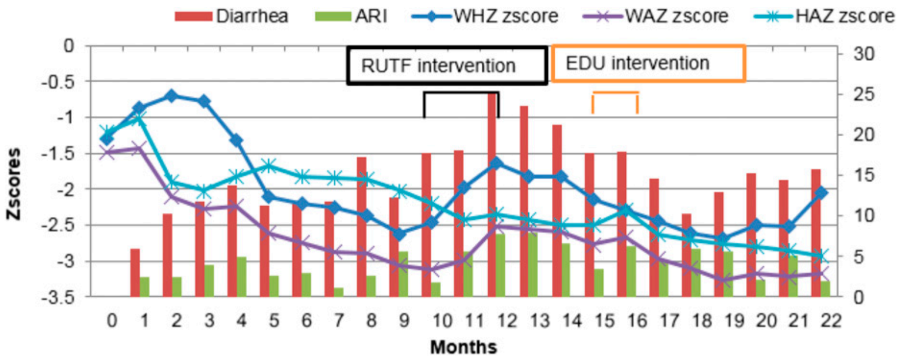

Supplemental Figure 2. Summary of respiratory and diarrheal morbidity data of $N = 10$ environmental enteropathy biopsy candidates. Data shown are average monthly episodes of ARI and diarrhea along with a trend in WHZ in our sample. ARI = acute respiratory infections; EDU = educational intervention; HAZ = height-for-height z score; RUTF = ready-to-use therapeutic food; WAZ = weight-for-height z score; WHZ = weight-for-height z score.

Supplemental Table 1

Clinical details on $N = 28$ patients with histopathologic examination showing normal duodenal tissue with no abnormality noted

Identification number	Gender	Age (months)	Race	Indication for endoscopy
1	M	12	White	Failure to thrive
2	F	12	White	Protein-losing enteropathy, poor per oral intake, and iron deficiency anemia
3	M	12	African American	Suspected esophageal reflux, egg allergy, and oral aversion
4	M	11	White	Gastroesophageal reflux disease and failure to thrive
5	M	12	African American	Failure to thrive
6	M	12	Hispanic	Failure to thrive and suspected intestinal malabsorption
7	M	12	African American	Food allergies and gastroesophageal reflux disease
8	M	12	White	History of tracheoesophageal fistula repaired at day of life 1 and gastroesophageal reflux disease
9	M	12	White	Vomiting
10	M	12	White	Esophageal reflux, a feeding problem, and suspected intestinal malabsorption
11	F	24	African American	Failure to thrive and weight loss
12	F	12	African American	H/o eosinophilic gastroenteritis, on steroids
13	F	8	African American	Follow-up of prior scope showing gastroesophageal reflux disease
14	F	12	Asian	Anemia
15	M	12	White	History of gastroesophageal reflux disease and multiple food allergies
16	M	12	White	Feeding problems
17	F	12	African American	Gastroesophageal reflux disease and nausea with vomiting
18	M	12	White	Diarrhea and weight loss
19	F	12	White	Eosinophilic esophagitis
20	F	12	White	Generalized abdominal pain, diarrhea, and weight loss
21	F	12	White	Suspected gastroesophageal reflux disease and diarrhea
22	M	12	White	Eosinophilic esophagitis
23	F	12	White	Diarrhea, nausea, and vomiting
24	M	12	White	Dysphagia
25	M	12	Unknown	Failure to thrive
26	M	36	African American	Infantile allergies to milk and peanuts, now with more abdominal pain
27	F	12	Unknown	History of oral aphthous ulcers
28	F	12	Unknown	Gastroesophageal reflux disease and poor weight gain

Supplemental Table 2

Clinical details on $N = 7$ patients with histopathologic examination showing duodenal tissue with non-celiac villous atrophy

Identification number	Gender	Age (months)	Race	Indication for endoscopy
1	M	12	Caucasian	Weight loss, vomiting, chronic diarrhea, and hypoalbuminemia
2	M	1	Unknown	Emesis and intractable diarrhea
3	M	24	Caucasian	Diarrhea, poor solid food intake, and weight loss
4	M	2.4	Unknown	Weight loss, anemia, and intractable diarrhea
5	M	10	Unknown	Recent hematemesis and GI bleeding
6	M	12	Asian	Hematochezia for several months, Clostridium difficile positive with perinuclear Anti-Neutrophil Cytoplasmic Antibodies positive
7	F	6	Hispanic	Feeding problems and intestinal malabsorption and undergoing follow-up upper GI endoscopy

Supplemental Table 3

Clinical details on N = 8 patients with histopathologic examination showing duodenal tissue with non-celiac lymphocytosis

Identification number	Gender	Age (months)	Race	Indication for endoscopy
1	F	6	White	Follow-up of prior endoscopy showing scattered eosinophils in the cardia biopsy and focal active inflammation in the antrum
2	F	19	Unknown	Oral aversion
3	M	12	African American	Vomiting and weight loss
4	F	12	Unknown	Failure to thrive, gastroesophageal reflux disease, and irritability
5	M	12	Unknown	History of feeding aversions, reflux, and poor weight gain
6	F	12	White	Vomiting and weight loss in children who are not tolerating feeds
7	M	12	White	Diarrhea and failure to thrive
8	F	12	White	Failure to thrive

Supplemental Table 4

Additional summary data from EE cases

	Total population median (Q1–Q3) or *n* (%)
EE cases characteristics at birth*	
Delivery	
Low birth weight < 2,500 g	5 (50%)
Preterm < 37 weeks GA	6 (60%)
Breastfeeding	10 (100%)
Home delivery	5 (50%)
Maternal	
Age	29 (3)
Literacy	0 (0%)
EE cases clinical characteristics at the time of biopsy	
Nutrition†	
Anemia	7 (70%)
Leukocytosis	2 (20%)
Iron deficiency	9 (90%)
B12 deficiency	1 (11%)
Folate deficiency	0 (0%)
D-25-OH deficiency	3 (33%)
D-25-OH insufficiency	5 (56%)
Celiac testing†	
Serum tTG-IgA—negative	9 (90%)
Serum IgA—normal	10 (100%)

EE = environmental enteropathy.

*Data from N = 10 EE biopsy cases.

†Definitions: anemia = Hb < 11.0 g/dL, leukocytosis: WBC count × 10^3/μL > 18.0; iron deficiency = serum ferritin < 15 μg/L or TSat% < 20; B12 deficiency = serum B12, pg/mL < 150; folate deficiency = serum folic acid < 3 ng/mL; vitamin D deficiency = 25-OH-D ng/mL < 20, insufficiency < 30; serum tTG IgA < 12 U/mL = negative; total serum IgA 0.18–1.7 g/L = normal. B12, folate and D-25-OH levels were available on N = 9 patients; N = 1 patient had equivocal levels of serum tTG IgA > 18 U/mL. GA = gestational age, preterm ≤ 37 weeks; Hb = hemoglobin; TSat = transferrin saturation; tTG IgA = anti-tissue transglutaminase immunoglobulin A; WBC = white blood count.

Supplemental Table 5

Duodenal aspirate pathogen burden for each biopsy subject N = 10

Identification	TaqMan PCR array on duodenal aspirates
1	*Giardia* spp. and *Helicobacter pylori*
2	*H. pylori*
3	*Enterovirus*, *Giardia* spp., and *H. pylori*
4	*Campylobacter* (*jejuni* and *coli*), *Giardia* spp., and *Norovirus* GII
5	Negative
6	*Giardia* spp.
7	*H. pylori*
8	Negative
9	Negative
10	Negative

No antibiotics were given to subjects 4–6 weeks before endoscopy.

Supplemental Table 6

Summary of stool TAC testing at 6 and 9 months of age for N = 10 biopsy subjects

Enteropathogens	6 months (Frequency of positive targets)	9 months (Frequency of positive targets)	6 months (% positive)	9 months (% positive)
Adenovirus	3	5	30	50
Adenovirus 40/41	2	1	20	10
Astrovirus	0	1	0	10
Enterovirus	3	4	30	40
Sapovirus	1	4	10	40
Enteroaggregative *Escherichia coli*_aatA	6	5	60	50
Enteroinvasive *E. coli/Shigella*	2	3	20	30
Enterotoxigenic *E. coli* - Labile Toxin	2	3	20	30
Enterotoxigenic *E. coli* - Stabile Toxin p	1	2	10	20
Enteropathogenic *E. coli*_bfpa	3	3	30	30
Enteroaggregative *E. coli*_aaiC	4	4	40	40
Enteropathogenic *E. coli*_eae	4	6	40	60
Giardia	4	3	40	30
Giardia triphosphate isomerase B	0	3	0	30
Norovirus GII	4	4	40	40
Campylobacter jejuni/coli	6	6	60	60
Campy pan	7	8	70	80
Cryptosporidium	2	3	20	30
Enterocytozoon bieneusi	1	2	10	20

TAC = TaqMan array card.

Supplemental Table 7

Helicobacter pylori and *Giardia* status in relation to frequency of $CD20^+$ B-cell aggregates

	H. pylori		*Giardia**	
	Positive	Negative	Positive	Negative
≥ 2 $CD20^+$ B-cell aggregates	3	4	0	5
< 2 $CD20^+$ B-cell aggregates	2	1	4	1

Statistical analysis (two-sided Fisher's exact test) confirmed that in the case of *H. pylori*, pathogen positivity and presence of ≥ 2 lymphoid aggregates did not co-occur more frequently than as expected by chance (P value not significant). In the case of *Giardia*, pathogen positivity and presence of ≥ 2 lymphoid aggregates co-occurred more frequently than expected by chance (P value < 0.05).

*Two-sided Fisher's exact test with P value < 0.05.
